# Quantification of electron accumulation at grain boundaries in perovskite polycrystalline films by correlative infrared-spectroscopic nanoimaging and Kelvin probe force microscopy

**DOI:** 10.1038/s41377-021-00524-7

**Published:** 2021-04-15

**Authors:** Ting-Xiao Qin, En-Ming You, Mao-Xin Zhang, Peng Zheng, Xiao-Feng Huang, Song-Yuan Ding, Bing-Wei Mao, Zhong-Qun Tian

**Affiliations:** 1 State Key Laboratory of Physical Chemistry of Solid Surfaces, Collaborative Innovation Center of Chemistry for Energy Materials, College of Chemistry and Chemical Engineering, Xiamen University, Xiamen, China; 2 School of Aerospace Engineering, Xiamen University, Xiamen, China

**Keywords:** Optical physics, Electronics, photonics and device physics

## Abstract

Organic–inorganic halide perovskites are emerging materials for photovoltaic applications with certified power conversion efficiencies (PCEs) over 25%. Generally, the microstructures of the perovskite materials are critical to the performances of PCEs. However, the role of the nanometer-sized grain boundaries (GBs) that universally existing in polycrystalline perovskite films could be benign or detrimental to solar cell performance, still remains controversial. Thus, nanometer-resolved quantification of charge carrier distribution to elucidate the role of GBs is highly desirable. Here, we employ correlative infrared-spectroscopic nanoimaging by the scattering-type scanning near-field optical microscopy with 20 nm spatial resolution and Kelvin probe force microscopy to quantify the density of electrons accumulated at the GBs in perovskite polycrystalline thin films. It is found that the electron accumulations are enhanced at the GBs and the electron density is increased from 6 × 10^19^ cm^−3^ in the dark to 8 × 10^19^ cm^−3^ under 10 min illumination with 532 nm light. Our results reveal that the electron accumulations are enhanced at the GBs especially under light illumination, featuring downward band bending toward the GBs, which would assist in electron-hole separation and thus be benign to the solar cell performance.

**Figure:**
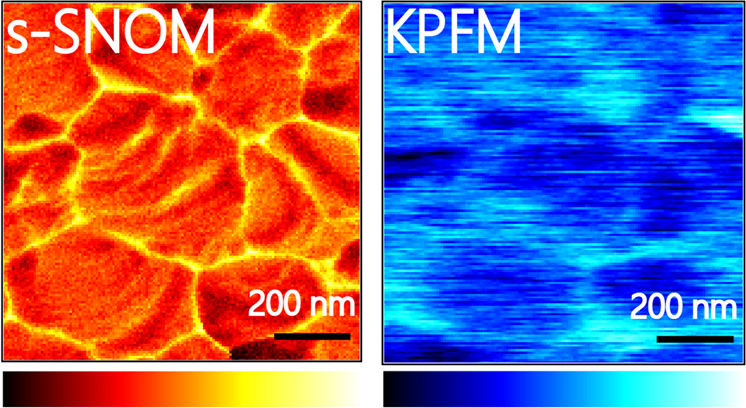
Correlative infrared-spectroscopic nanoimaging by the scattering-type scanning near-field optical microscopy and Kelvin probe force microscopy quantitatively reveal the accumulated electrons at GBs in perovskite polycrystalline thin films.

Correlative infrared-spectroscopic nanoimaging by the scattering-type scanning near-field optical microscopy and Kelvin probe force microscopy quantitatively reveal the accumulated electrons at GBs in perovskite polycrystalline thin films.

## Introduction

Organic–inorganic halide perovskites (e.g., CH_3_NH_3_Pb*X*_3_, *X* = Cl, Br, I), featuring large absorption coefficient, high carrier mobility, and long diffusion length^1–5^, become emerging materials for solar cells with rapidly boosted power conversion efficiency (PCE) from 3.8% in 2009^6^ to a recently certified over 25%^7^. Though different synthetic methods such as spin coating^8–10^, thermal coevaporation^11^, vapor-assisted depositions^12^, have been developed to pursue good crystalline morphology with high uniformity, the inherent polycrystalline nature of perovskite active layers leads to unavoidable existence of a large number of grain boundaries (GBs), similar to inorganic solar cells materials^13–15^. Understanding the role of GBs of polycrystalline perovskite thin films in solar cell performance is crucial for the rational design of perovskite active layer structures.

However, the issue about whether the GBs in a perovskites polycrystalline thin-film solar cell is electrically benign or detrimental to solar cell performances has triggered many theoretical and experimental studies. First-principle calculations suggest that while the intrinsic GBs in inorganic solar cells thin films, such as GaAs and Cu(In, Ga)Se_2_, generate deep levels states in band gaps and are therefore harmful for the device performance^16^, the GBs in the CH_3_NH_3_PbI_3_ films with shallow point defects are electrically benign and are beneficial to the PCE of the perovskites solar cells^17,18^. However, this result is contrary to the conclusions obtained by nonadiabatic molecular dynamics studies combined with time-domain density functional theory calculations that GBs have negative influences owing to the accelerated electron-hole recombinations in CH_3_NH_3_PbI_3_^19,20^. The first-principle calculations show that the enhanced structural relaxation of the defects at GBs results in the accumulations of deep traps (faster recombination)^21^. Experimental observations are also contradictory. For example, it has been demonstrated by nanoscale imaging techniques such as Kelvin probe force microscopy (KPFM)^22–25^ that GBs bear higher surface potential with smaller work functions. A classical model^26^ of interfacial states suggests that owing to the downward band bending toward the GBs, a barrier is formed with the built-in potential. As a result, the GBs would repel holes and attract electrons, which is expected to increase the minority-carrier (electrons) collection at the GBs and can be beneficial to the photovoltaic performance. Moreover, the spatially resolved imaging on photocarrier generations by scanning tunneling microscopy also indicates that efficient charge separation occurs at the heterointerface of grains^27^. However, this conclusion is contradictory with the observations that large grain sizes with reduced GBs possess better solar cell performances as reported in literatures^28–30^, which implies that GBs accelerate electron-hole recombination and thus degrade the optoelectronic properties of the film. In addition, the correlating microscopy measurements with scanning electron analytical techniques further consider that the presence of the surface trap states at grain junctions would limit the device performance^31^. In view of the debates on the current reports, it is necessary to quantitatively measure the local free-carrier density to deeply understand the role of GBs^32^.

KPFM and conducting atomic force microscope (c-AFM) are widely used to provide information about surface potential and integral current, respectively, at the perovskite GBs at the nanoscale, but they fail to directly quantify the distribution of the free-carrier densities. On the other hand, based on the Drude-like free-carrier absorption in the mid-infrared (IR), which occurs as the carrier density of samples is ~10^19^~10^20^ cm^−3^,^33–37^, scattering-type scanning near-field optical microscopy (s-SNOM) has been applied to quantify the carrier density distributions of highly *p*-doped poly-Si^33,34,36,38^, InP nanowires^35^, zinc oxide nanowires^37^, and doped SrTiO_3_ ceramics^39^. In s-SNOM, a metal-coated AFM tip, under the illumination of a focused IR laser beam, is used to further concentrate the IR light into a nanoscale region underneath the tip apex for strengthening the near-field interaction between the nanoscale IR light and the sample. Thus, near-field amplitude and phase signals are acquired by demodulating the backscattered light from the metalized AFM tip working in a tapping mode^36^. Analyzing the amplitude and phase signals based on the Drude model allows for quantifying the carrier density distributions.

Here, we employ the s-SNOM imaging method to quantify the spatial distribution of carrier densities at GBs and intragrains (IGs) in the polycrystalline perovskite thin-film. Taking CH_3_NH_3_PbI_3_ as an example, larger near-field amplitudes at GBs rather than at IGs were measured, revealing higher carrier density at GBs. Quantitative analysis of the enhanced near-field amplitude under 532 nm laser illumination further shows that the density of carriers accumulated at the GBs increase from 6 × 10^19^ cm^−3^ to 8 × 10^19^ cm^−3^ in the perovskite layer. Correlative nanoimaging of s-SNOM and KPFM further shows that larger near-field amplitudes and higher surface potentials are more localized at the GBs, suggesting the accumulation of electrons due to the downward band bending at the GBs in perovskite polycrystalline films. The electron accumulation behavior of GBs in perovskite active layers can assist in electron-hole separations, which is benign to the solar cell performance.

## Results

### Broadband s-SNOM image

**Fig. 1:**
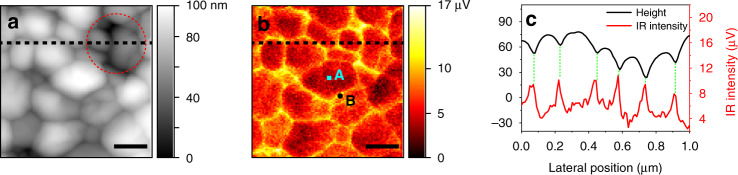
Infrared near-field images recorded on a CH_3_NH_3_PbI_3_/FTO/glass sample. **a** Topography image (1 μm × 1 μm). **b** Corresponding broadband infrared near-field image, with the reference mirror fixed at position *d* ≈ 0. Positions A and B indicate the regions of IG and GB, respectively. **c** One-dimensional near-field amplitude and the topography line profiles along the black dashed lines in **a** and **b**. The optical signal intensity was recorded by the mercury-cadmium-telluride (MCT) detector so that signal in the unit of V was used. The scale bars in **a** and **b** are 200 nm

First, near-field imaging was performed to qualitatively analyze the distribution of carriers in the perovskite film by the s-SNOM with a broadband laser source that covers the wavenumber range from 650 to 1400 cm^−1^. The AFM topography image (Fig. 1a) of a polycrystalline film surface of CH_3_NH_3_PbI_3_ on an FTO/glass (CH_3_NH_3_PbI_3_/FTO/glass) shows the sizes of the grains range from 100 to 200 nm with height variations of ~20 nm (see the line profiles shown in Fig. 1c). The simultaneously acquired infrared near-field amplitude image in Fig. 1b, featuring 20 nm spatial resolution as analyzed in Figure S1, exhibits a strong contrast between GBs and IGs: GBs appear brighter with larger amplitudes, whereas IGs are darker with smaller amplitudes. Such a contrast can be further analyzed by correlating the surface topography (Fig. 1a) with the corresponding one-dimensional near-field amplitude (Fig. 1b) along the black dashed line. As shown in Fig. 1c, the near-field amplitudes have strong contrast between the GBs and IGs. However, they also have additional correlations, though weak, with the locations of the surface, which can be seen from the anticorrelation between the lg(IR intensity) and lg(GB gap size) as shown in Figure S2^40,41^. Thus, it is necessary to clarify whether the larger infrared signals at GBs originate from the enhanced carrier distribution or from a topography-induced infrared image difference.

**Fig. 2:**
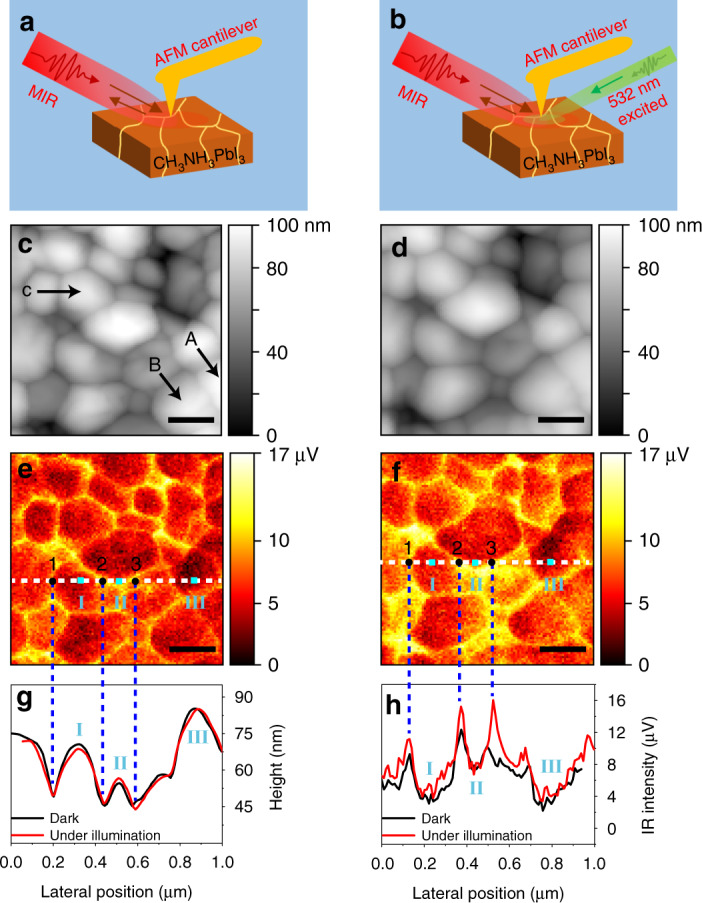
Topography and infrared near-field image in the dark and under illumination. **a** Schematic illustration of the s-SNOM setups in the dark and **b** with external illumination of a 532 nm laser; **c** AFM topography, and **e** the simultaneously acquired infrared near-field image in the dark; **d** AFM topography and **f** infrared near-field image in the same area under the illumination by 532 nm laser for 10 min. **g** and **h** are the one-dimensional topography and near-field amplitude line profiles along the white dashed lines in **e** and **f**, respectively. Three locations of GBs are marked by black dots and numbered as 1, 2, 3 in **e** and **f**, whereas the cyan solid squares labeled by I, II, III mark three locations of IGs. The scale bars for all the images are 200 nm

To evaluate the possibility of the topography-induced enhancement of the infrared signals at GBs, we compared the variation of the near-field amplitude in the depression region (marked with a red circle in Fig. 1a) with those in other regions. As shown in Fig. 1c, although the depression region is ~30 nm lower than the nearby regions, the near-field amplitudes remain comparable in these regions. The ratios of the variations of height and near-field amplitude at various GBs are not fixed (Figure S3b), which means that there is no one-to-one relationship between the height and near-field signal^36^. Further analysis of another line profile of the correlative one-dimensional near-field amplitude and the topography was shown in Figure S4. Therefore, the larger infrared signals at GBs are not completely derived from topographical effect. In fact, the infrared near-field amplitude in s-SNOM is related to the near-field interaction between the localized infrared and free carriers (plasmons) in the sample^33,36,42–44^. As a result, the carrier accumulation at the GBs would result in the large near-field amplitude at the GBs of the perovskite films^23,25,27^. Moreover, the near-field amplitude signals increase at the grain boundary when the visible light is turned on (see below Fig. 2h), which is associated with the increase in carrier density. Thus, the illumination experiment further confirms that the carrier accumulation contributes to infrared image contrasts.

It has been reported that the chemical composition signatures of perovskite could also contribute to the near-field spectra^45^. But, we did not observe the specific absorption peak of MA^+^, which might be owing to the weaker infrared response of the N-H bending in MA^+^^46^ compared with the C-N stretching in FA^+^^45^. Probably, the interference of chemical composition signatures is minimized in this study. In addition, the ions could accumulate at the interfaces under an externally applied bias^47^. However, no external bias was applied in this study. Thus, the chemical signatures moieties and ionic accumulation of the MAPbI_3_ films could not contribute to the near-field signals.

### Broadband s-SNOM image under 532 nm laser illumination

The carrier distribution at the surface of the perovskite film can be tuned by the visible illumination^24,25,27,43,48^. The infrared near-field image of the perovskite film under light illumination was conducted to probe the density and spatial distribution of photocarriers at GBs and IGs in the film. As shown in Fig. 2b and Figure S5, visible laser centered at 532 nm was used to excite more free carriers in the perovskite films. The AFM topography in Fig. 2d (under 10 min of illumination) remains almost unchanged, compared with the one in the dark conditions in Fig. 2c. However, the infrared near-field amplitudes in the illuminating condition (Fig. 2f) become stronger. The enhanced near-field signals can be further verified through the line profile analysis shown in Fig. 2g, h. Specifically, the near-field amplitudes at the GBs are enhanced from 9.3, 12.3, and 10.3 µV (black line) to 11.3, 15.3, and 16.1 µV (red line) at positions 1, 2, and 3 marked in Fig. 2f, respectively, under 10 mins illumination. Three other GBs located at different positions (marked as A, B, C in Fig. 2c) also show the enhanced near-field amplitudes when the perovskite films are under visible light illumination (see the details analysis in Figure S6). However, the amplitude signals at the IGs recorded at positions I, II, and III are rarely influenced. The significantly enhanced near-field amplitude at the GBs indicates that more carriers are accumulated and trapped in the GBs under the 532 nm laser illumination. It is noted that the enhanced carrier density under illumination is temporary (Figure S7) and the time-dependent measurements show that the s-SNOM signal intensity of the illuminated sample increases as prolonged illumination time (Figure S8).

### Near-field spectra of perovskite polycrystalline film at GBs and IGs

**Fig. 3:**
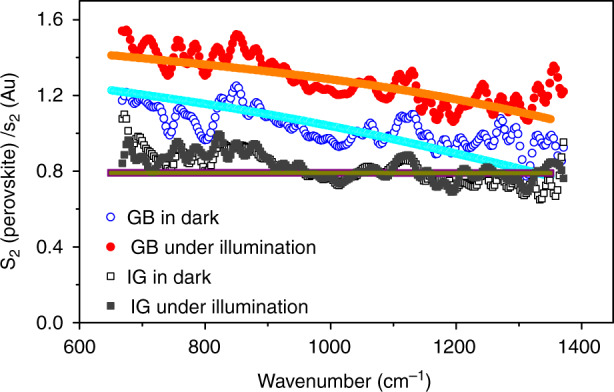
Near-field spectra *s*_2_(perovskite)/*s*_2_(Au) of CH_3_NH_3_PbI_3_ polycrystalline film (*s*_2,IG_/*s*_2,Au_ at position A and *s*_2,GB_/*s*_2,Au_ at position B marked in Fig. 1b). The solid lines show calculations performed with the finite dipole model of s-SNOM, using a probe with tip-radius of 25 nm and tip-tapping amplitude of 60 nm. The charge mobility and dielectric data for CH_3_NH_3_PbI_3_ are taken from the literatures;^2,51^ and the free-carrier density *n* is the fitting parameter. The best agreement between the calculations and the experiments on CH_3_NH_3_PbI_3_ was found for a free-carrier density *n* = 6 × 10^19^ cm^−3^ at GB in the dark and *n* = 8 × 10^19^ cm^−3^ at GB under 532 nm laser illumination

To quantify the carrier accumulation at the GBs under the 532 nm laser illumination, broadband near-field spectra were recorded, as shown in Fig. 3. The near-field spectra at positions IG (A) and GB (B) marked in Fig. 1b are normalized to that of Au. In the dark, the amplitude spectra at GB (blue open circle) decrease gradually as the wavenumber increases from 650 to 1400 cm^−1^, and the spectra at IG (gray open square) show weaker intensities than those at the GBs. The near-field spectra can be assigned to the near-field interaction between the tip and free carriers (plasmons) in the perovskite film^36^. The free-carrier absorption below 1500 cm^−1^ has been directly demonstrated by the time-resolved infrared spectroscopy in CH_3_NH_3_PbI_3_^49^. Moreover, a finite dipole model of s-SNOM^50^ was employed to interpret the near-field spectra, in which the near-field interaction between the probing tip and perovskite film was described by their dielectric functions, *ε*_Au_ and *ε*(CH_3_NH_3_PbI_3_), respectively (see details of the model calculation in Supplementary Note 2). The calculated near-field spectra of GB (cyan solid lines) and IG (purple solid lines) in the dark are in agreement with the experimental data, with the fitting carrier density of *n* = 6 × 10^19^ cm^−3^ and *n* = 1 × 10^16^ cm^−3^, respectively.

The near-field amplitude spectrum of IG under the external illumination of the 532 nm laser is similar to that in the dark condition (Fig. 3). The invariance of the amplitude at the IGs upon laser illumination can also be seen from the line profiles shown in Fig. 2h. The near-field amplitude spectrum of the GB (red closed circle) increases by ~20% compared with that in the dark. The calculated near-field amplitude spectrum (orange solid line) of the GB under illumination is in good agreement with the experimental data, with a fitting carrier density of *n* = 8 × 10^19^ cm^−3^. Spectral calculations show that the carrier density located at the GBs increases from 6 × 10^19^ cm^−3^ in the dark to 8 × 10^19^ cm^−3^ under 532 nm laser illumination, which indicates the accumulation of carriers in the GBs under external light illumination. In summary, the broadband near-field spectral investigation has provided the quantification of the spatial distribution of carrier densities at the GBs.

### Correlative nanoimaging of s-SNOM and KPFM

**Fig. 4:**
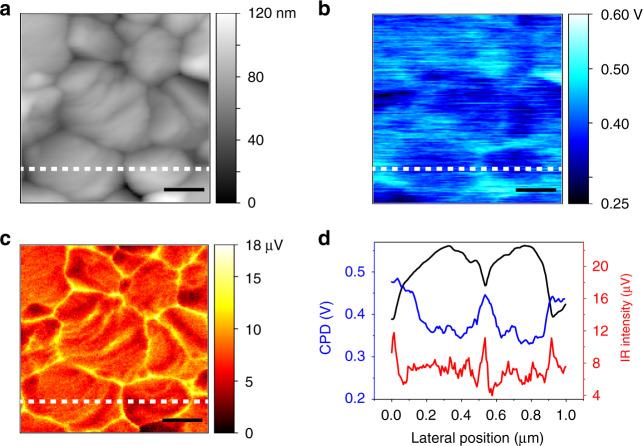
Correlative KPFM and s-SNOM nanoimaging on perovskite. **a** AFM topography (1 μm × 1 μm); **b** Contact potential difference (CPD); and **c** simultaneously acquired infrared near-field image; **d** one-dimensional line profiles of the topography, CPD and infrared near-field amplitude along the white dashed lines marked in **a**–**c**. The scale bars are 200 nm

To gain insights into the relationships between the electrical properties and spectral information at GBs, integrated KPFM and s-SNOM measurements were performed to acquire the surface potential and infrared near-field image simultaneously through a single-pass scan^52,53^. Figure 4a displays the topography of the perovskite polycrystalline film with a grain size of 200–500 nm. A clear contrast of the surface potential is observed around the GBs in the contact potential difference (CPD) image (Fig. 4b), which implies the difference in the electrical property. As shown in Fig. 4d, the higher CPD at GBs indicates that the local built-in potential is formed due to the downward band bending at GBs, which is consistent with previous reports, indicating the electron accumulation at GBs^22,23,25^. This conclusion can also be deduced from the correlative broadband s-SNOM image (Fig. 4c) and line profile (Fig. 4d) of the near-field amplitude which appears to be brighter with larger amplitude intensity at GBs. Furthermore, the line profiles (Fig. 4d) also show that the full width at half maximum of amplitude at the GBs is much narrower than that of the CPD in the KPFM measurement, indicating a much higher spatial resolution of s-SNOM (27 nm) with respect to that of KPFM (57 nm) as analyzed in Figure S9. The correlative broadband s-SNOM and KPFM measurements, which were performed simultaneously, are meaningful on a qualitative level despite the spatial resolution of s-SNOM mismatches that of KPFM.

**Fig. 5:**
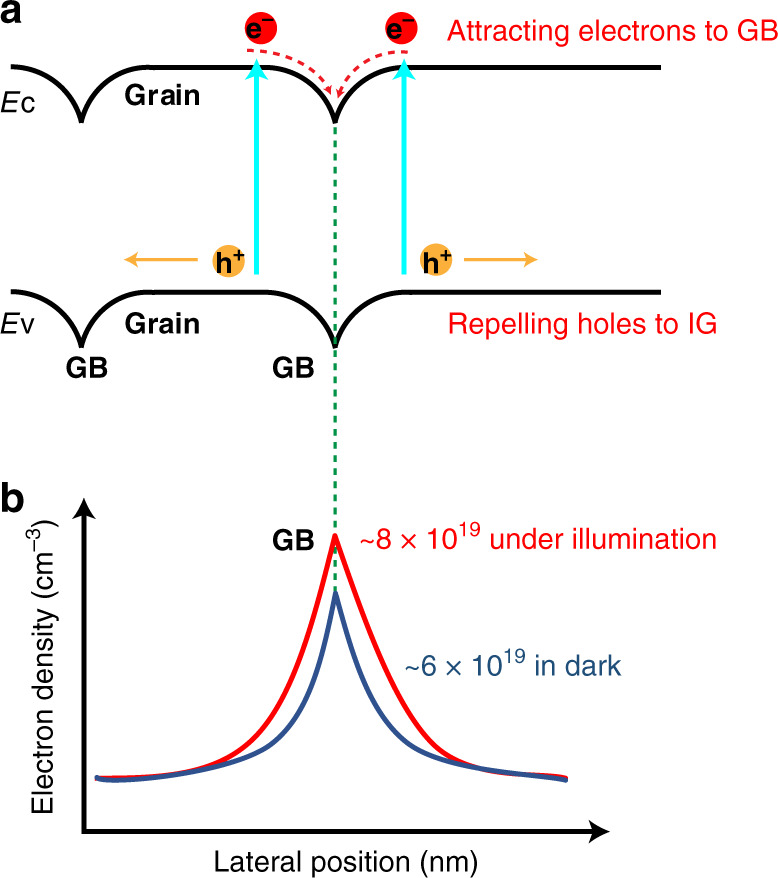
Physical picture illustration of the electron accumulations at GBs. **a** Schematic illustration of the band alignment between IGs and GBs. The yellow/red solid circles indicate holes/electrons, and the yellow/red arrows show their direction of movement. *E*_C_ and *E*_V_ stand for the conduction band bottom and the valance band top, respectively. **b** Schematic illustration of electron density around the GB with and without additional light illumination. The downward band bending at the GB results in an accumulation of electrons in the GB region with electron density increases from 6 × 10^19^ cm^−3^ to 8 × 10^19^ cm^−3^ (solid red line) under 532 nm illumination

The correlative KPFM and s-SNOM measurements help us unambiguously understand the physical picture at the GBs of the CH_3_NH_3_PbI_3_ polycrystalline films. As indicated in Fig. 5a, the local built-in potential leads to the attraction of the electrons to the GBs and repelling of the holes to the IGs, assisting in electron-hole carrier separation and thus suppressing the recombination. Moreover, it has been reported that the local built-in potential also leads to the polarity inversion in the space-charge region around the GBs from inherent *p*-type grain bulk^30,54^ to n-type with the electron densities ~6 × 10^19^ cm^−3^ at the GBs. Our results also support the inverted GB polarity in inorganic solar cells around the GB reported for the research on Cu(In, Ga)Se_2_ films^55,56^. Under externally visible illumination, photoinduced electrons populated at the conduction band, further accumulate at the GBs owing to built-in potential, leading to increased electron density from 6 × 10^19^ cm^−3^ in the dark to 8 × 10^19^ cm^−3^ under the illumination, as illustrated in Fig. 5b. This physical picture presented in Fig. 5 supports the models proposed in previous reports^22–25,57^.

## Discussion

In conclusion, we have quantified the electron accumulation behavior at the GBs in polycrystalline CH_3_NH_3_PbI_3_ perovskite films by employing infrared near-field imaging technique (s-SNOM) with a spatial resolution of 20 nm. Broadband s-SNOM images indicate large near-field amplitude at the GBs, implying the higher carrier density at the GBs in a polycrystalline perovskite layer. Moreover, the results from broadband s-SNOM images further reveal that the carrier density increases from 6 × 10^19^ cm^−3^ in the dark to 8 × 10^19^ cm^−3^ under 532 nm laser illumination. Correlative nanoimaging of s-SNOM and KPFM further indicates the electron accumulation behavior of GB in perovskite active layers, elucidating the relationship between the spectral information and the electrical properties. Our observations by infrared nanoimaging with correlative KPFM are in accordance with previously reported KPFM and c-AFM results, and further confirm that the downward band bending at the GBs assists in electron-hole carrier separation and thus suppresses recombination, which would be benign to solar cell performance.

The correlative broadband s-SNOM and KPFM measurements can be extended to other perovskite structures with vibrational fingerprint information in the infrared spectra, such as FAPbI_3_ (FA = formamidinium, a main molecular absorption emerges at ~1700 cm^−1^, which is attributed to antisymmetric C-N stretching of the FA molecule)^45^. In this case, the chemical information of individual grains on their local composition can be measured by broadband s-SNOM spectroscopy. Revealing the relationship between the electrical properties and the chemical compositions of perovskite at the nanoscale can potentially disclose how the interrelations of both would affect the device performance and further guide how to rationally design the perovskite active layer structures.

## Materials and methods

### Room-temperature SSE deposition of perovskite polycrystalline films

PbI_2_ and CH_3_NH_3_I were purchased from Xi’an Polymer Light Technology Corp. (PLT). All of the reagent-grade chemicals were used as received. CH_3_NH_3_PbI_3_ perovskite polycrystalline films were prepared according to the reported solvent–solvent extraction (SSE) method as described in literature^58^. In brief, a 42 wt% solution of PbI_2_ and CH_3_NH_3_I (molar ratio 1:1) in 1﻿-Methyl-2-pyrrolidinone (NMP) was prepared. A 30 μL 42 wt% CH_3_NH_3_PbI_3_ solution was spin-coated onto fluorine-doped tin oxide (FTO) coated glass substrates (TEC 15), and then, the solution was spun at 4500 rpm for 30 s. The solution-coated substrate was vertically dipped in a ~50 ml anhydrous Diethyl ether (DEE) bath immediately. The substrate was kept immersed until a brown film formed in ~2 min. The substrate was then taken out of the bath and transferred to a hotplate at 150°C covered by a petri dish for 15 min with air annealing. The entire perovskite film fabrication process was performed at ambient conditions with ~35% humidity.

### Infrared s-SNOM measurements

A commercialized neaSNOM system (neaspec, GmbH), based on s-SNOM, was utilized to perform s-SNOM IR imaging^33,35–37^. Standard Pt/Ir probes (Arrow-NCPt, Nanoworld) with a resonance frequency of Ω ≈ 250 kHz were used in the experiments, and the tapping amplitude of the cantilever system was ~60 nm. A broadband MIR laser, covering from 650 to 1400 cm^–1^, is generated by a difference frequency generator (TOPTICA Photonics AG). The MIR laser is focused by the parabolic mirror to concentrate the IR light into a nanoscale region underneath the tip apex. For the broadband s-SNOM image, the reference mirror in the s-SNOM system was kept fixed at position *d* ≈ 0 (*d* denotes the optical path difference between the light backscattered from the tip and reflected from the reference mirror in the asymmetric Michelson interferometer), which corresponds to the white light position. At this position, the beam path lengths are equal for both the signal and reference arm, which gives the strongest signal owing to constructive interference under which the interference of all spectral components simultaneously maximizes the detector signal intensity. The second-order demodulation method was employed for efficient background suppression. Nano-FTIR spectra were obtained by constantly moving the mirror in the reference arm of the Michelson interferometer, recording the resulting interferograms and their corresponding complex Fourier transformation.

For the illumination experiments, the sample was illuminated with a wavelength of 532 nm (corresponding to an energy of 2.33 eV, which is larger than the bandgap of perovskite) by a 10 mW solid-state laser. The 532 nm laser spot focused with the parabolic mirror below the tip position was elliptical with major and minor axes of (60 ± 5) μm and (40 ± 5) μm, which was determined with a microscope. The estimated irradiation power intensity at the sample surface was ~200 mW/cm^2^, which was controlled by a variable neutral-density filter wheel.

### KPFM

Correlative s-SNOM and KPFM were also measured by a commercialized neaSNOM system (neaspec, GmbH). We chose PPP-EFM probes (Nanosensors) whose fundamental resonance frequency was 78.5 kHz. The KPFM measurements were performed with a single-pass scan utilizing a frequency-modulation mode. The surface potential responses were extracted from the second eigenresonance of the probe. The feedback d.c. bias voltage was applied to the tip. Thus, *e* × *V*_CPD_ = *Φ*_tip_ − *Φ*_sample_, where *Φ*_tip_ and *Φ*_sample_ are the work functions of the tip and sample, respectively.

## Supplementary information

SUPPLEMENTAL MATERIAL

## Data Availability

The data supporting the findings of this study are available from the corresponding authors upon request.
