# Supplementary material for: Quantification of electron accumulation at grain boundaries in perovskite polycrystalline films by correlative infrared-spectroscopic nanoimaging and Kelvin probe force microscopy
Source: Light Sci Appl. 2021 Apr 15;10:84. doi: 10.1038/s41377-021-00524-7 (PMC8050298; doi:10.1038/s41377-021-00524-7)
Supplement: Supplementary file 1 — SUPPLEMENTAL MATERIAL [file 41377_2021_524_MOESM1_ESM.docx]

Supplementary Information for

Quantification of Electron Accumulation at Grain Boundaries in Perovskite Polycrystalline Films by Correlative Infrared-spectroscopic Nanoimaging and Kelvin Probe Force Microscopy

For the Special Issue on the 100th Anniversary of Xiamen University.

*Ting-Xiao Qin,^1,#^ En-Ming You,^1,#^ Mao-Xin Zhang,^1^ Peng Zheng,^2^ Xiao-Feng Huang,^1^ Song-Yuan Ding,^1,*^ Bing-Wei Mao,^1,*^ Zhong-Qun Tian^1^*

^1^ State Key Laboratory of Physical Chemistry of Solid Surfaces, Collaborative Innovation Center of Chemistry for Energy Materials, College of Chemistry and Chemical Engineering, Xiamen University, Xiamen 361005, China

^2^ School of Aerospace Engineering, Xiamen University, Xiamen 361005, China

**# Equal contributions * Corresponding authors**

**Correspondence:**

Song-Yuan Ding: syding@xmu.edu.cn, +86-0592-2186979

Bing-Wei Mao: bwmao@xmu.edu.cn, +86-0592-2186862


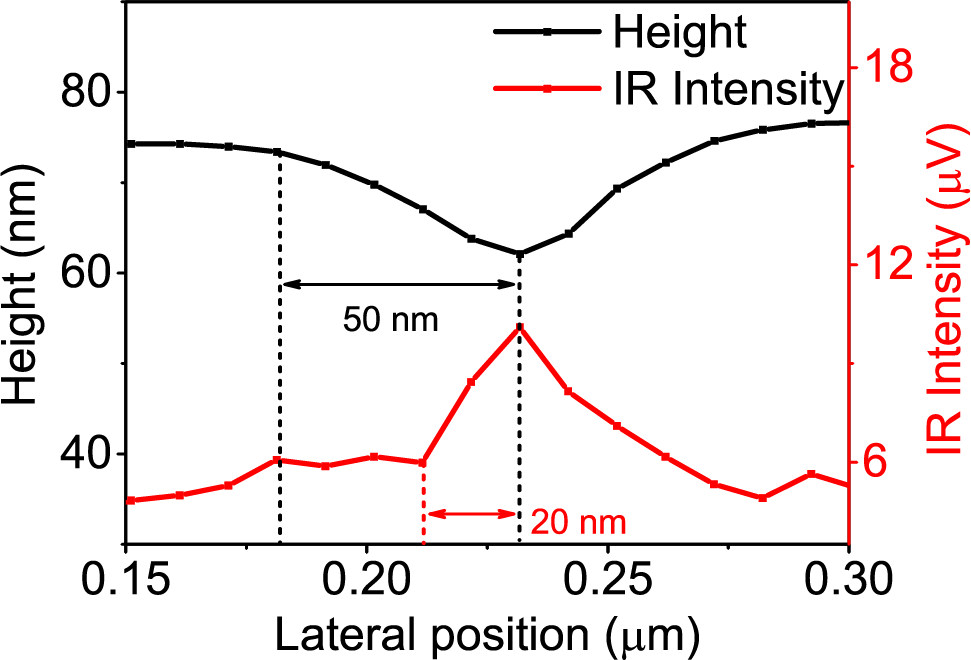


**Figure S1.** The spatial resolution of s-SNOM at one of the GBs. The spatial resolution of s-SNOM can be estimated from a section profile in Figure 1b, showing a spatial resolution of 20 nm. The width of the height change is about 50 nm, which is due to the intrinsic sloping of GBs edges.


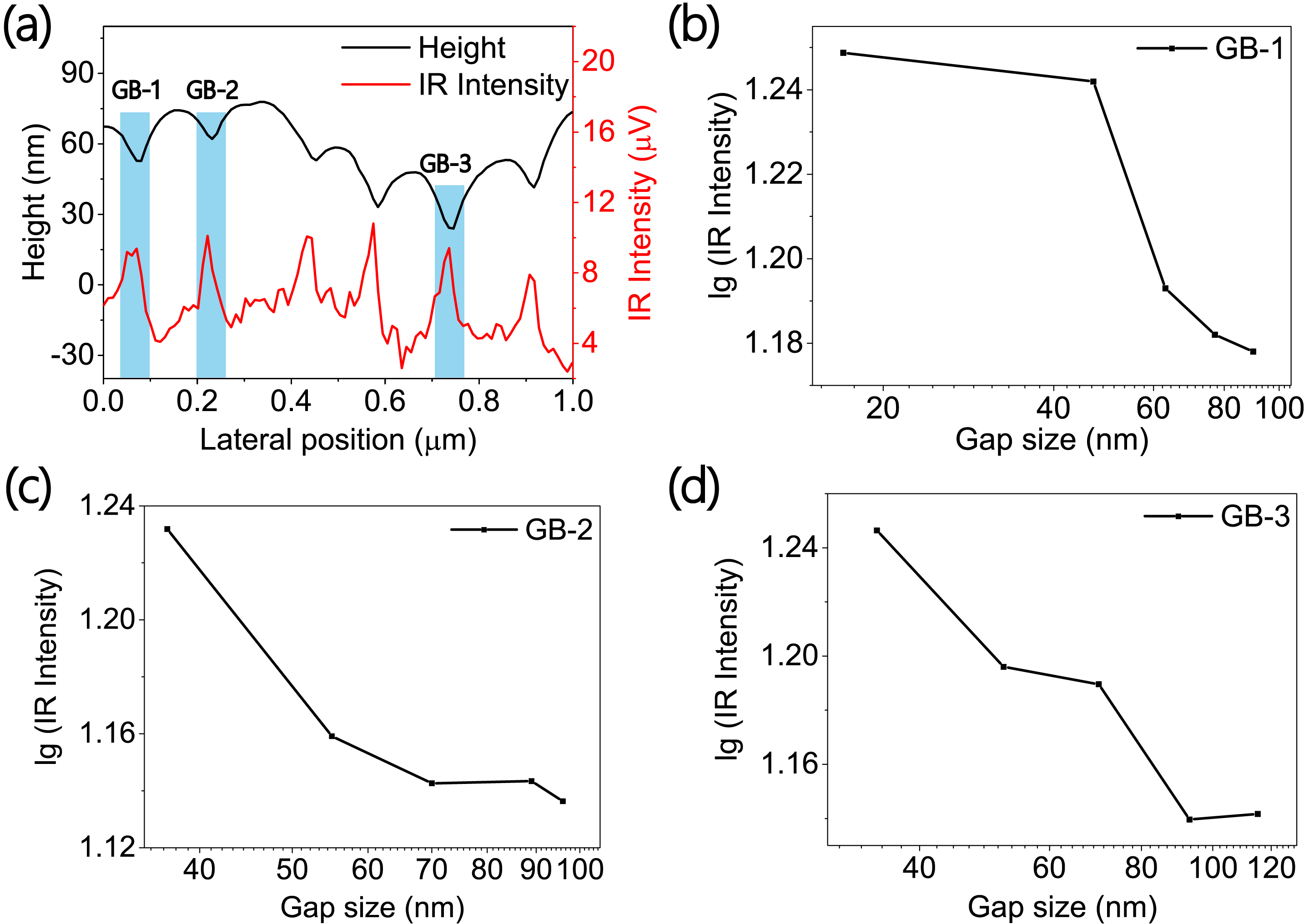


**Figure S2.** The relationship between the near-field intensity and the GB gap sizes.

We identified three GBs located at different positions (marked as GB-1, GB-2, GB-3 in Figure S2a) for further analysis of the relationship between the near-field intensity and the GB gap sizes. Figure S2b, S2c, S2d show that the near-field intensity increased while the GB gap sizes narrowed^1, 2^.


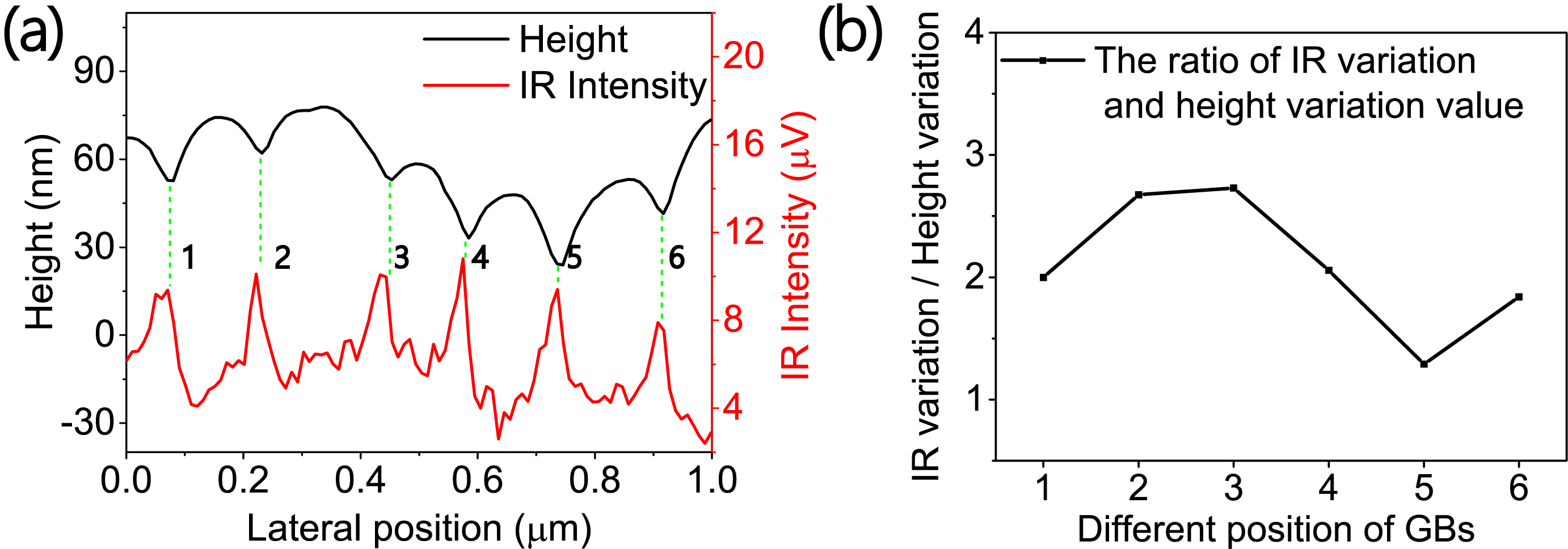


**Figure S3.** (a) One-dimensional near-field intensity and the topography line profiles in the perovskite thin films; (b) The ratios of the IR variation/Height variation caused by GBs with different 6 points marked in Figure S3a.


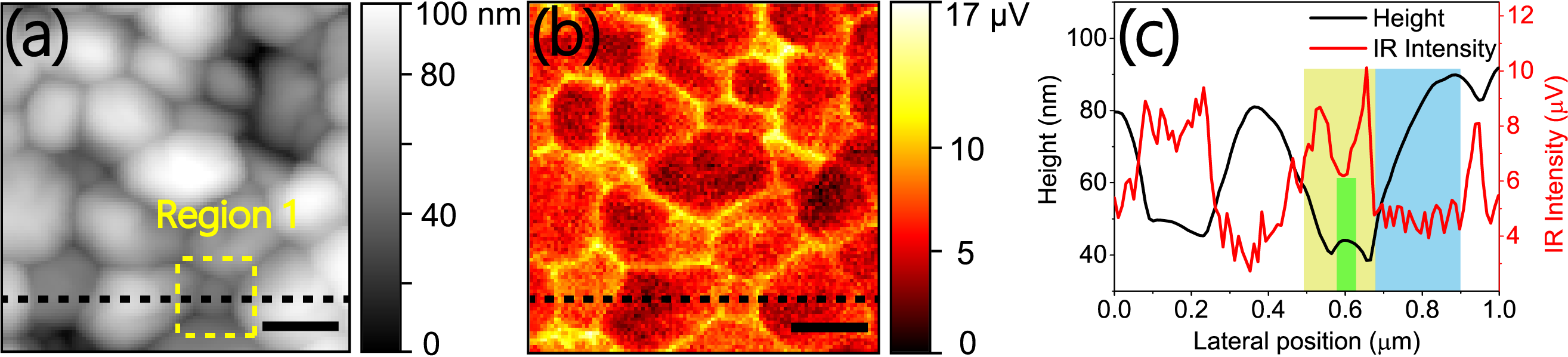


**Figure S4.** (a) Topography image (1 *μ*m × 1 *μ*m); (b) Infrared near-field image of the corresponding topography; (c) One-dimensional near-field amplitude and topography line profiles. The region of the line profiles is marked as a black dash line in (a) and (b). The scale bars in (a) and (b) are 200 nm.

Another correlative one-dimensional near-field amplitude and the topography line profiles along the black dash lines in Figure S4a and S4b are shown in Figure S4c. In the depressed Region 1, the height difference caused by the GBs is smaller than the sunken depth (as marked by the green region in Figure S4c), but the changes in the near-field amplitudes at the GBs are larger than the depressed regions. Furthermore, at the lateral position between 0.7 and 0.9 *μ*m (the blue region in Figure S4c), the height changes substantially but there is almost no change in the infrared intensity. These results indicate that there is no one-to-one correspondence between the height and the infrared signals.


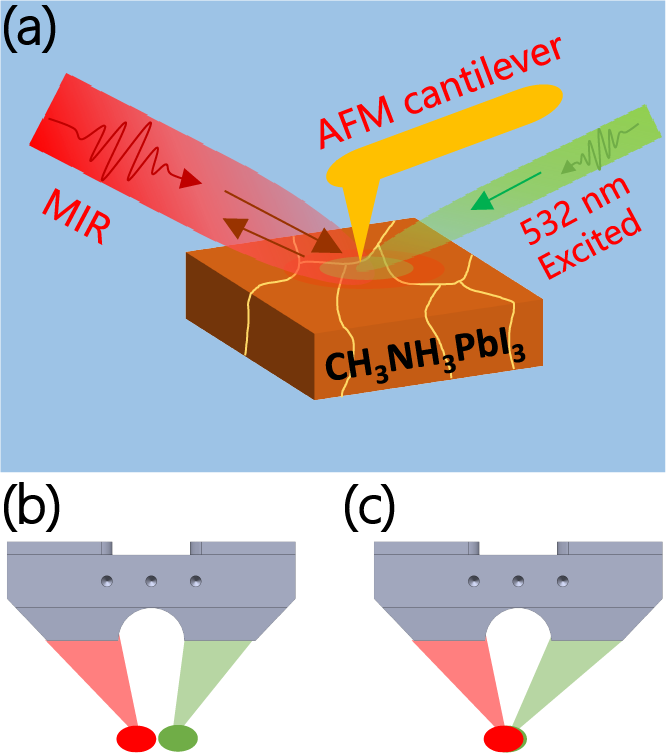


**Figure S5.** Schematic illustration with additional side illumination.

To excite more carriers in perovskite polycrystalline films, an additional visible laser centered at 532 nm is used, whose photon energy can excite photocarriers in perovskite. We expect to see different contrast in the optical image, which can be resolved to be the change in carrier density. The schematic diagram is illustrated in Figure S5. In our neaSNOM system, the 532 nm visible laser and the MIR probe laser are incorporated into the system from opposite directions. The visible excitation laser illuminated via the right side, and the mid-infrared measuring laser illuminated from the left side, as shown in Figure S5a. In general, the systems allow very slight misalignment, which leads to different working points for the right and the left side (Figure S5b). We must carefully optimize the parabolic mirror to overlap these two laser points (Figure S5c).


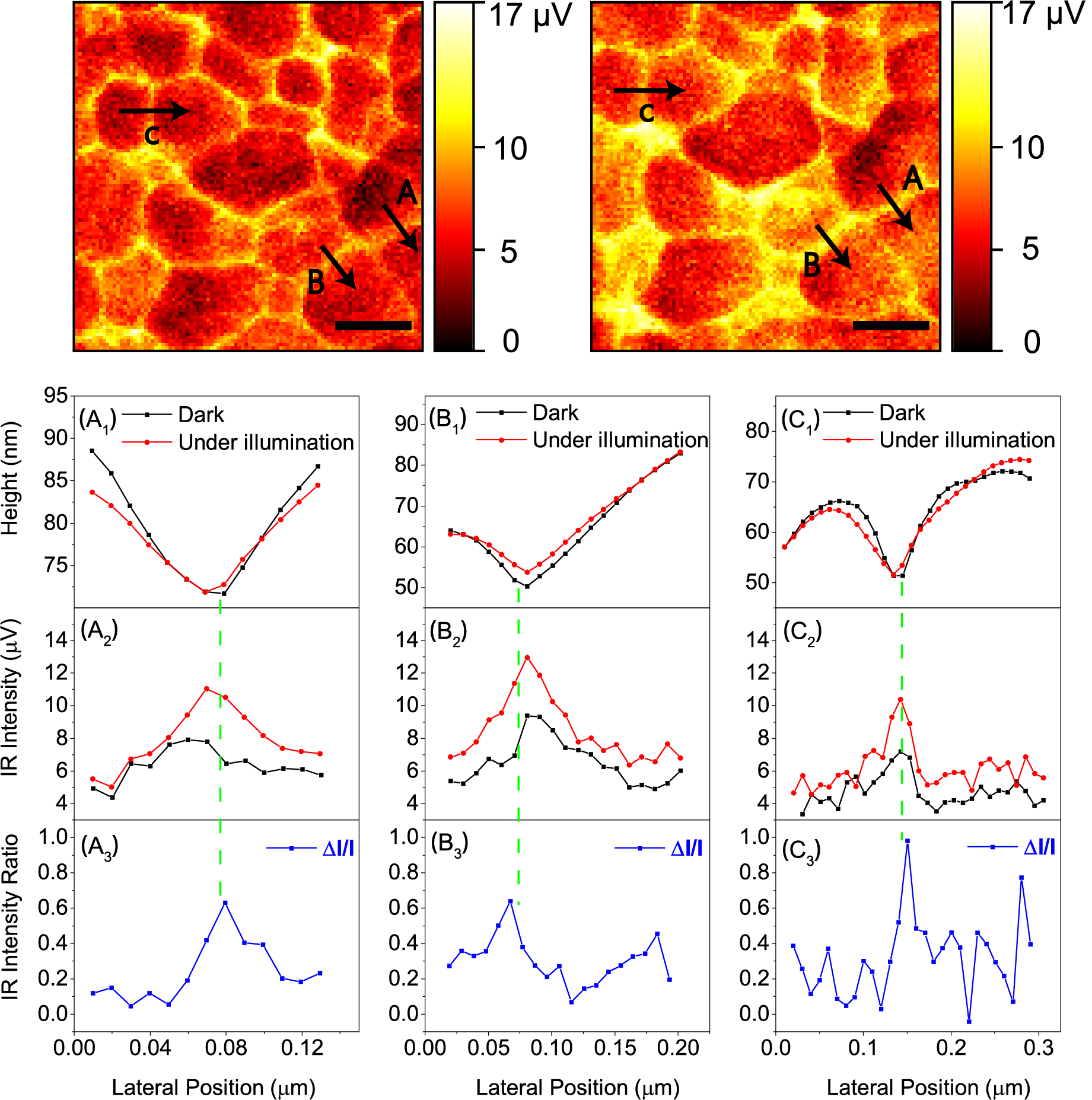


**Figure S6.** Positions A, B, C marked in the infrared near-field image. The arrows indicate the lateral locations of the line profiles of the topography and near-field images. The scale bars are 200 nm. (A_1_, B_1_, C_1_) One-dimensional topography line profiles; (A_2_, B_2_, C_2_) One-dimensional near-field intensity line profiles in dark and with 532 nm laser illumination for 10 min; (A_3_, B_3_, C_3_) Near-field intensity enhancement ratios between GBs and IGs. We define ΔI/I = (illumination–dark)/dark as a near-field amplitude intensity enhancement factor to compare the differences in the near-field amplitude enhancement between the GBs and IGs under external illumination. As shown in Figure S6 A3, B3, C3, the enhancement factors at the GBs (~0.6) are larger than that at the IGs (~0.2) under external illumination. The significantly enhanced near-field amplitude at the GBs indicates that more carriers are accumulated in the GBs under the 532 nm laser illumination.


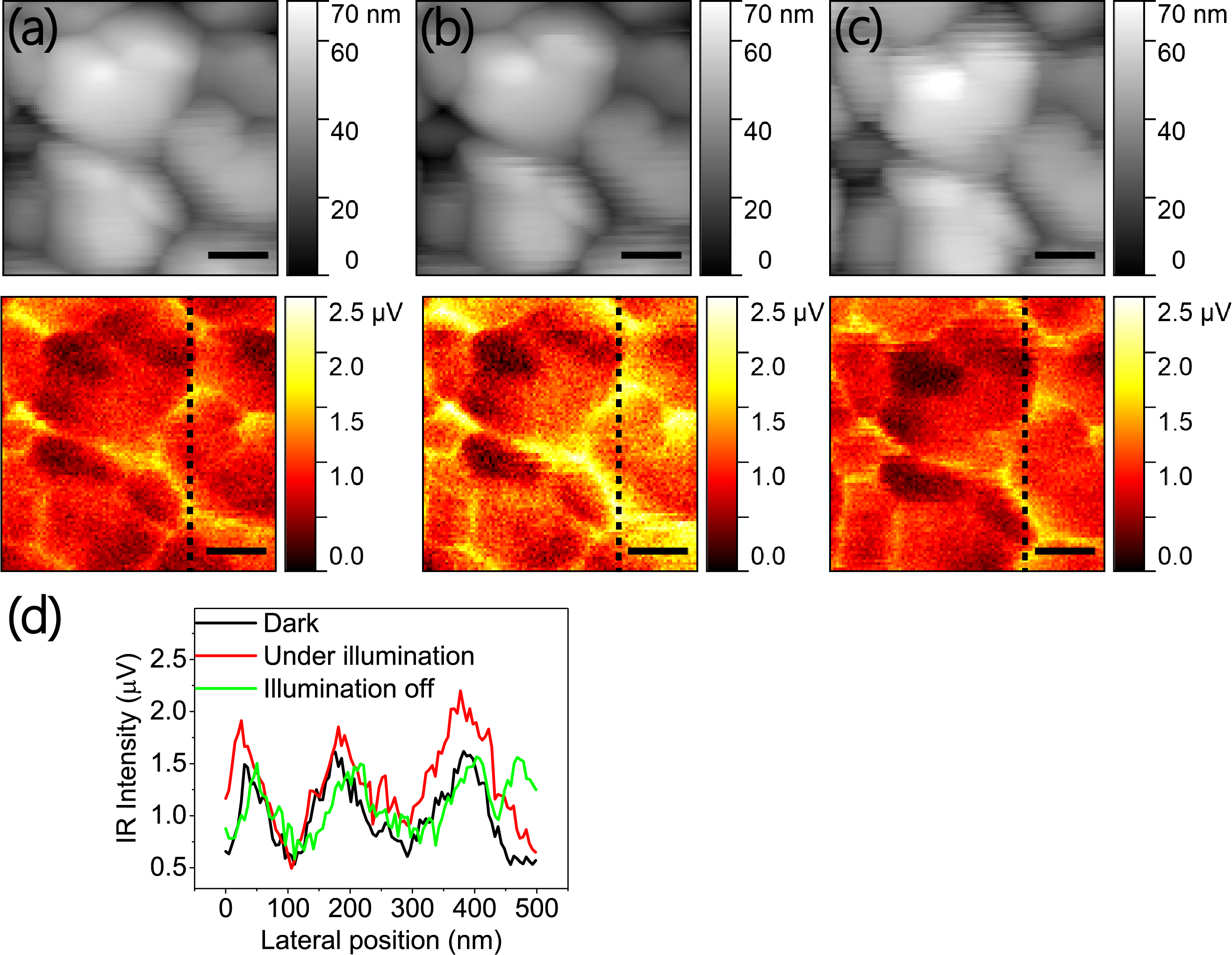


**Figure S7.** Temporary change of the carrier density. (a) AFM topography and the simultaneously acquired infrared near-field image without the 532 nm laser; and (b) at the same area under 10 min illumination; (c) with the 532 nm laser off. The scale bars are 100 nm. The infrared near-field amplitudes increase when the 532 nm laser was turned on (Figure S7b) in contrast to the amplitudes in the dark and the laser turned off (Figure S7c). The time-dependent variation of near-field signals can be seen through the line profile analysis in Figure S7d, indicating the temporarily enhanced carrier density under illumination.


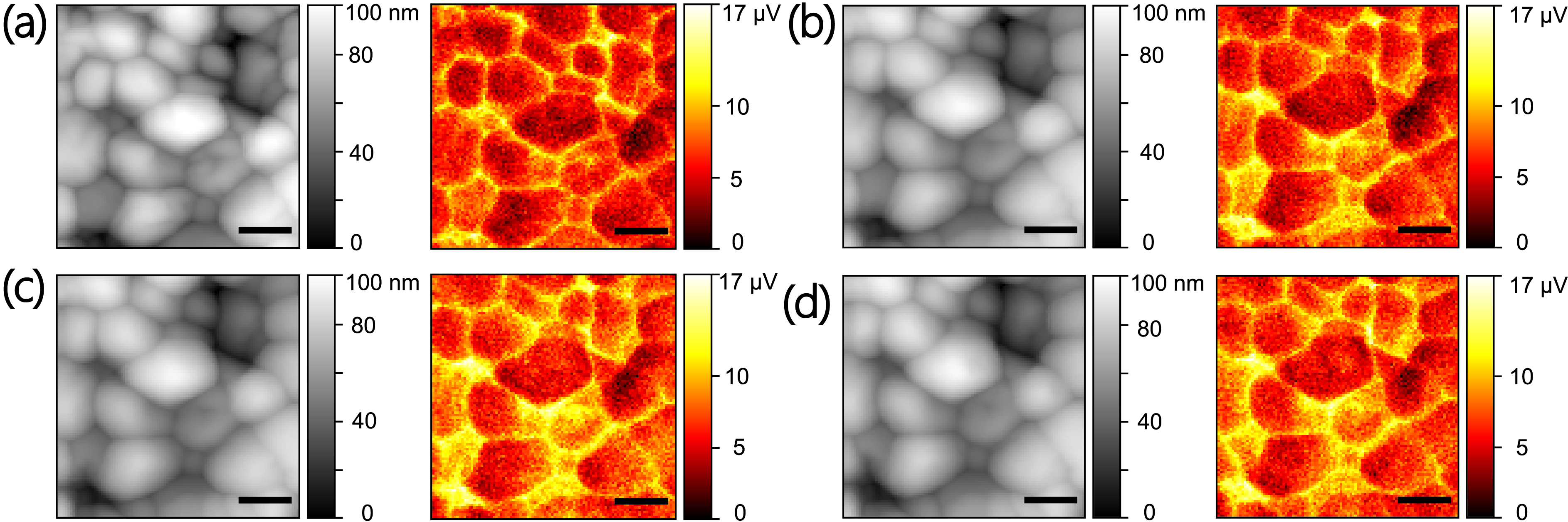


**Figure S8.** Time-dependent s-SNOM measurements. (a) AFM topography and the simultaneously acquired infrared near-field image without 532 nm laser; and infrared near-field image at the same area after different illumination time with 532 nm laser (b) 5 min; (c) 10 min; (d) 15 min. The scale bars are 200 nm. The s-SNOM signal intensity of illuminated sample increases as prolonged illumination time, which can be used to elucidate the enhanced carrier density at the GBs with increasing input of total laser energy at a qualitative level.


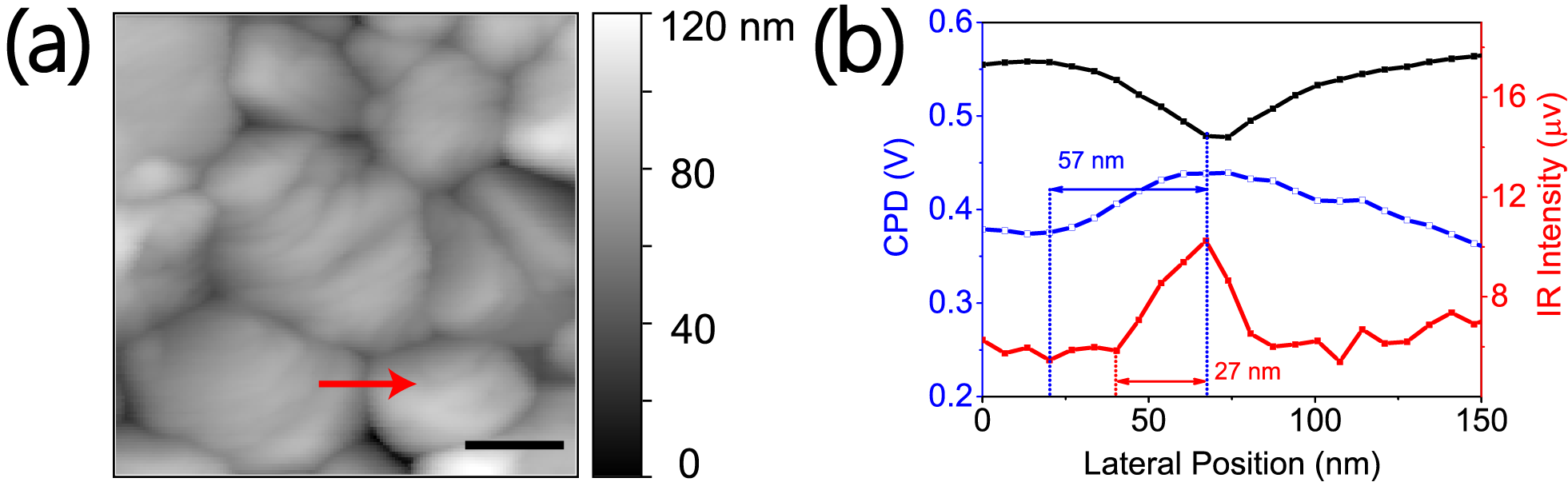


**Figure S9.** Spatial resolution of s-SNOM is shown to be 27 nm, which is better than KPFM determined to be 57 nm. The scale bar is 200 nm.

Supplementary Note 1. Experimental details

Our IR s-SNOM technique is based on atomic force microscopy (AFM) as described previously^3-6^. The tip is illuminated with a focused laser beam. Due to their metallization and sharp apex, these tips act like an antenna and concentrate the illuminating electric field 𝐸_in_ to a nanoscale spot at their apex. The “lightning rod” effect known from electrostatics combined with the excitation of surface plasmons polaritons in the metal, which gives rise to very high local fields. When the illuminated tip comes in close proximity to a sample, the near-field interaction between tip and sample modifies the light scattered by the tip. The near-field interaction can be described by the complex valued scattering coefficient 𝜎 = 𝑠𝑒^𝑖𝜑^, from which near-field amplitude (*s*) and phase spectra (*φ*) are obtained. The near-field scattering coefficient 𝜎 that relates the light backscattered from the tip 𝐸_sca_ with the incoming light 𝐸_in_, according to 𝐸_sca_ = 𝜎 𝐸_in_. The scattered field contains unavoidable background contributions from other parts of the AFM cantilever or the sample surface outside the tip region (𝐸_bg_), yielding the total scattered field 𝐸_sca_ = 𝐸_nf_ + 𝐸_bg_. To suppress the background signal, the tip is oscillating at the mechanical resonance of AFM cantilever in tapping mode and perform lock-in demodulation or Fourier analysis on scattered light at a non-fundamental harmonic of the tip oscillation frequency^4, 7^. The *n*^th^ order demodulated near-field amplitude and phase are denoted as *s*_n_ and *φ*_n_. In this article, we use the 2^th^ order demodulated signal to eliminate the background signal and preserve the near-field signal. Therefore the background-free near-field signals can be obtained. The near-field signals directly reflect the local permittivity of the sample underneath the AFM tip, providing access to distinguish the nanometer-resolved dielectric properties of materials. The detailed description between the tip and the sample can be understood through the finite dipole model^8^, which is related the complex valued near-field scattering coefficient 𝜎 to the dielectric properties of the sample. The theoretical models are discussed in great detail in ref.^5,6,8^.

Supplementary Note 2. Finite-Dipole Model Calculation

Theoretical s-SNOM near-field spectra at the GBs and IGs are calculated by using the finite-dipole model^8^. In this model, amplitude signals are derived from the near-field interaction between the probing tip and the bulk sample, as characterized by its dielectric function $\varepsilon_{\mathrm{CH}_{3}\mathrm{NH}_{3}\mathrm{PbI}_{3}}$^9^. The presence of free carriers is taken into account by a Drude-term, yielding a dielectric function of CH_3_NH_3_PbI_3_ given by

$$\varepsilon\left( \omega\right)=\varepsilon_{\infty}-\frac{\omega_{p}^{2}}{\omega^{2}-ⅈ\Gamma\omega}$$

where $\omega_{p}^{2}=\frac{nⅇ^{2}}{\varepsilon_{0}m_{e_{ff}}}$

where ω_p_ denotes the plasma frequency, which depends on the free carrier concentration *n* according to ω_p_^2^ ∼ *n*. The damping parameter Γ is directly linked to the mobility of the charge carriers μ, which is Γ=e[2πc m_eff_ μ]^–1^. Here, m_0_ is the electron mass, m_eff_ is the effective electron (hole) mass^10^, and ε_∞_ is the high-frequency dielectric constant of CH_3_NH_3_PbI_3_, which is adopted from the literature^9^.

The reported charge mobility values in perovskite bulk single crystals are approximately 10 s to 100 cm^2^ V^–1^ s^–1 11-13^, while for polycrystalline films, they are different by orders of magnitude (10^–5^ to 10 s cm^2^ V^–1^ s^–1^)^14, 15^, which has been partially attributed to the heterogeneous distribution of the grain size, grain boundary, and defects. However, in another microscope research study on neat CH_3_NH_3_PbI_3_(Cl) polycrystalline perovskite films, the local (intra-grain) carrier diffusivities in different grains are all comparable to bulk single crystals^16^. Due to the mobility μ differing by order of magnitude, we assume in the model electron mobility values of ~25 cm^2^ V^–1^ s^–1^, as taken from ref.^11^ deduced from CH_3_NH_3_PbI_3_ single crystals.

In our calculations, the calculation parameters use the tapping amplitude A = 60 nm and tip radius R = 25 nm; both values were taken from the experiment. The free-carrier concentration *n* is the fit parameter. No other fit parameter has been applied. All of the calculation parameters are summarized in Table S1.

**Table S1.** The calculation parameters used in the finite-dipole model.

| ε^∞9^ | m_eff_ (m_e_*)^10^ | m_eff_ (m_h_*)^10^ | μ (cm^2^ V^–1^ s^–1^)^11^ | Amplitude  (nm) | Tip radius (nm) |
| --- | --- | --- | --- | --- | --- |
| 7 | 0.23 | 0.29 | 25 | 60 | 25 |

4. References

1. Hatab, N. A. *et al.* Free-standing optical gold bowtie nanoantenna with variable gap size for enhanced Raman spectroscopy. *Nano Letters* **10**, 4952-4955 (2010).

2. Huck, C. *et al.* Surface-enhanced infrared spectroscopy using nanometer-sized gaps. *ACS Nano* **8**, 4908-4914 (2014).

3. Hillenbrand, R., Taubner, T. & Keilmann, F. Phonon-enhanced light-matter interaction at the nanometre scale. *Nature* **418**, 159 (2002).

4. Ocelic, N., Huber, A. & Hillenbrand, R. Pseudoheterodyne detection for background-free near-field spectroscopy. *Applied Physics Letters* **89**, 101124 (2006).

5. Ocelić, N. Quantitative near-field phonon-polariton spectroscopy. *PhD Thesis*, (2007).

6. Huth, F. Nano-FTIR nanoscale infrared near-field spectroscopy. *PhD Thesis*, (2015).

7. Knoll, B. & Keilmann, F. Enhanced dielectric contrast in scattering-type scanning near-field optical microscopy. *Optics Communications* **182**, 321-328 (2000).

8. Cvitkovic, A., Ocelic, N. & Hillenbrand, R. Analytical model for quantitative prediction of material contrasts in scattering-type near-field optical microscopy. *Optics Express* **15**, 8550-8565 (2007).

9. Juarez-Perez, E. J. *et al.* Photoinduced giant dielectric constant in lead halide perovskite solar Cells. *The Journal of Physical Chemistry Letters* **5**, 2390-2394 (2014).

10. Giorgi, G., Fujisawa, J.-I., Segawa, H. & Yamashita, K. Small photocarrier effective masses featuring ambipolar transport in methylammonium lead iodide perovskite: A density functional analysis. *The Journal of Physical Chemistry Letters.* **4**, 4213-4216 (2013).

11. Dong, Q. *et al.* Electron-hole diffusion lengths >175 *μ*m in solution-grown CH_3_NH_3_PbI_3_ single crystals. *Science* **347**, 967-970 (2015).

12. Shi, D. *et al.* Low trap-state density and long carrier diffusion in organolead trihalide perovskite single crystals. *Science* **347**, 519-522 (2015).

13. Tian, W. *et al.* Visualizing carrier diffusion in individual single-crystal organolead halide perovskite nanowires and nanoplates. *Journal of the American Chemical Society* **137**, 12458-12461 (2015).

14. Shao, Y. *et al.* Origin and elimination of photocurrent hysteresis by fullerene passivation in CH_3_NH_3_PbI_3_ planar heterojunction solar cells. *Nature Communications* **5**, 5784 (2014).

15. Xiao, Z. *et al.* Efficient, high yield perovskite photovoltaic devices grown by interdiffusion of solution-processed precursor stacking layers. *Energy & Environmental Science* **7**, 2619-2623 (2014).

16. Tian, W. *et al.* Limiting perovskite solar cell performance by heterogeneous carrier extraction. *Angewandte Chemie International Edition* **55**, 13067-13071 (2016).
